# Plasma-Wind-Assisted In_2_S_3_ Preparation with an Amorphous Surface Structure for Enhanced Photocatalytic Hydrogen Production

**DOI:** 10.3390/nano12101761

**Published:** 2022-05-21

**Authors:** Shaohui Guo, Hui Luo, Xiaochuan Duan, Bingqing Wei, Xianming Zhang

**Affiliations:** 1 College of Chemistry, Taiyuan University of Technology, Taiyuan 030024, China; guoshaohui@tyut.edu.cn (S.G.); duanxiaochuan@tyut.edu.cn (X.D.); 2 Department of Chemical Engineering, Imperial College London, South Kensington Campus, London SW7 2AZ, UK; hui.luo@imperial.ac.uk; 3 Department of Mechanical Engineering, University of Delaware, Newark, DE 19716, USA; 4 Key Laboratory of Interface Science and Engineering in Advanced Material, Ministry of Education, Taiyuan University of Technology, Taiyuan 030024, China

**Keywords:** photocatalytic hydrogen evolution, crystal–amorphous interface, plasma-wind treatment, indium sulfide

## Abstract

Photocatalytic production from water is considered an effective solution to fossil fuel-related environmental concerns, and photocatalyst surface science holds a significant interest in balancing photocatalysts’ stability and activity. We propose a *plasma-wind* method to tune the surface properties of a photocatalyst with an amorphous structure. Theoretical calculation shows that the amorphous surface structure can cause an unsaturated coordination environment to adjust the electron distribution, forming more adsorption sites. Thus, the photocatalyst with a crystal–amorphous (C–A) interface can strengthen light absorption, harvest photo-induced electrons, and enrich the active sites, which help improve hydrogen yield. As a proof of concept, with indium sulfide (In_2_S_3_) nanosheets used as the catalyst, an impressive hydrogen production rate up to 457.35 μmol cm^−2^ h^−1^ has been achieved. Moreover, after plasma-assisted treatment, In_2_S_3_ with a C–A interface can produce hydrogen from water under natural outdoor conditions. Following a six-hour test, the rate of photocatalytic hydrogen evolution is found to be 400.50 μmol cm^−2^ g^−1^, which demonstrates that a catalyst prepared through plasma treatment is both effective and highly practical.

## 1. Introduction

Overusing fossil fuels is the primary cause of global environmental problems and the energy crisis. Photocatalytic hydrogen production from water is a promising green route to address such issues. With its high energy density, hydrogen gas is a clean replacement for fossil fuels [1,2,3,4,5]. However, a significant obstacle to the industrialization of photocatalytic hydrogen production is low conversion efficiency. Many strategies have been therefore proposed to enhance the optical, electrical, and catalytic properties of photocatalysts to improve their efficiency, including heterojunction structural design [6,7], the introduction of plasmonic materials [8,9,10], defect engineering (e.g., oxygen or sulfur vacancy) [11,12], phase transition [13,14], and single-atom catalysis [15,16,17]. It is worth noting that catalysis is a surface redox reaction on the photocatalyst, where the water molecules split into hydrogen and oxygen [18]. Therefore, it makes sense to tune and improve the surface structure of photocatalysts to enhance their reactive interface with water molecules. However, some photocatalysts’ surface structure can usually be adversely affected in the liquid water environment, degrading catalytic stability and the chemical reaction. Thus, any industrially promising photocatalyst for hydrogen evolution reaction should balance material stability and activity with a harmonized surface structure design.

We report a *plasma-wind* strategy to realize the “surface amorphous engineering” to improve photocatalysts’ surface structure. A photocatalyst with a crystalline–amorphous (C–A) interface can boost light absorption and accelerate charge transfer [19,20,21]. In this work, In_2_S_3_ nanosheets are used as a typical catalyst with plasma treatment to obtain a tunable amorphous surface structure. In_2_S_3_ is a standard IIIA-VIA semiconductor that can be prepared through sol–gel or hydrothermal methods. This nanosheet has been researched as a visible-light-driven photocatalyst for water splitting and degradation [22,23,24,25]. Based on experimental observations and theoretical calculations, the coordination environment in the In_2_S_3_ nanosheet has been tuned through the plasma treatment. Thus, plasma-treated In_2_S_3_ (P-In_2_S_3_) with rich active sites has been achieved. Furthermore, considering the convenience of this plasma treatment, we have tested a practical model under natural outdoor conditions for further development, which displays the potential of this technique for industrialization.

## 2. Materials and Methods

### 2.1. Synthesis of the In_2_S_3_

FTO glass was pre-cleaned through ultrasonic cleaning in ultrapure water and ethanol. After that, FTO glass was further treated with UV cleaning. InCl_3_ (0.05 g), thioacetamide (0.05 g), and the treated FTO glass were added into a Teflon-lined stainless steel autoclave. Then, 40 mL of ultrapure water was added to obtain a mixed solution. The autoclave was heated at 150 °C (about 0.4 MPa) for 6 h and cooled to room temperature naturally. Hereafter, the resulting samples were named In_2_S_3_.

### 2.2. Synthesis of the P-In_2_S_3_

The prepared In_2_S_3_ in FTO was placed in the plasma-treatment device (AC220-2g). The Ar gas was the carrier gas, and the time was set for 60 min. The power was 200 W. The chamber pressure was 200 Torr, and the Ar flow rate was 50 cc min^−1^. The sample was placed 25 mm away from the powered electrode. Then, after the reaction, the P-In_2_S_3_ was obtained.

### 2.3. Characterization of the Photocatalysts

The morphology and structure of the samples were characterized using a scanning electron microscope (SEM) (FEI NOVA 450, FEI Company, Hillsboro, OR, USA) and transmission electron microscope (TEM) (FEI Talos F200X). The absorption spectrum was measured using an ultraviolet-visible (UV-Vis) spectrophotometer (Perkin-Elmer Lambda 35 UV-VIS-NIR, Waltham, MA, USA). The electrochemical workstation (Chenhua 760e, CH Instruments, Shanghai, China) was used for the photoelectric performance test. The EIS at a frequency range from 100 kHz to 1 Hz was measured. The Raman spectra were collected through the Renishaw with the 532 nm laser. During the catalytic measurements, the Raman spectra were collected at the same interval voltage to eliminate the extra influence induced through bias voltage (the measurement voltage was 0.05, 0.00, −0.05, −0.10, and −0.15 V) under the same light intensity (5 mW), and the exposure time was 5 s.

### 2.4. Photocatalytic Activity

The photocatalytic reaction was conducted by photocatalysts (1 cm^−2^) with 50 mL of deionized water in a quartz cell, loaded with 0.3 M Na_2_S and 0.3 M Na_2_SO_3_ as hole scavengers. A light source was equipped with a fin-like heat sink for dissipating excessive heat effectively. The incident power was measured by a power meter. The average power was determined to be 100 mW cm^−2^. The gases evolved during the photocatalytic reaction and were transferred into a sample loop by a peristaltic pump. They were further quantified using gas chromatography (Shimadzu GC-2014c (Kyoto, Japan), Ar carrier gas, and molecular sieve-5A column), equipped with a thermal conductivity detector (TCD) with a set temperature of 422 K. The yield of hydrogen gas produced from the reactor was measured every 15 min. For the cyclic test, the reactor is replenished with 0.3 M Na_2_S and 0.3 M Na_2_SO_3_ and degassed in a vacuum before starting the irradiation for another measurement.

### 2.5. Computational Details

All calculations presented here were implemented in the Vienna ab initio simulation package (VASP 5.4, Hafner Group, Wien, Austria) [26]. The generalized gradient approximation (GGA) in Perdew–Burke–Ernzerhof (PBE) format was used for the exchange-correlation function [27]. The long-range van der Waals interaction was described by the DFT-D3 approach [28]. The cut-off energy for the plane wave was set at 400 eV. The energy and force convergence criteria for structure relaxations were 0.1 meV/atom and 0.03 eV/Å, respectively. The geometry was optimized with a 3 × 3 × 1 k-mesh grid.

The bulk In_2_S_3_ constituted 80 atoms, the lattice parameters were obtained by fully relaxing the structure, and the obtained *a* and *c* were 7.74 Å and 32.85 Å, respectively. Amorphous In_2_S_3_ structure was constructed by density functional theory molecular dynamics (DFT-MD). The atoms in the crystal structure were melted at 2000 K for 7000 MD steps and quenched from 2000 to 300 K for 7000 MD steps [29]. A surface model of In_2_S_3_ in (002) orientation was created based on the optimized crystal and amorphous bulk geometry. A vacuum region of 15 Å was added perpendicular to the sheet to avoid artificial interaction between periodic images; the bottom layers of the slabs were fixed during relaxation.

The free energies were calculated by treating the H adsorbate within the harmonic oscillator approximation, which is given by ΔG=ΔE +ΔZPE −TΔS, where ΔZPE and ΔS are the changes in the zero-point energy and entropy, respectively, and T is the temperature of 298.15 K.

## 3. Results and Discussion

### 3.1. The Synthesis Process and Related Morphological, Spectroscopic Characterization

**Figure 1:**
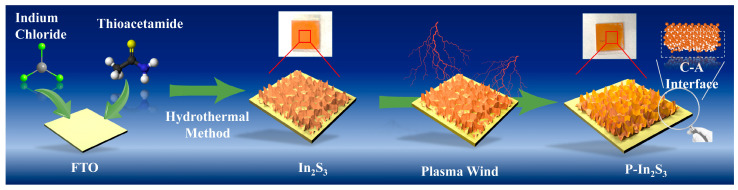
The schematic of the fabrication process of the sample P-In_2_S_3_.

As shown in Figure 1, indium chloride and thioacetamide are used to prepare the In_2_S_3_ nanosheets. The nucleation event of In_2_S_3_ nanosheets takes place on the surface of fluorine-doped tin oxide (FTO) glass. The In_2_S_3_ nanosheets are distributed uniformly on the surface of the FTO glass, and the assembly is then subject to plasma treatment. Thus, the plasma-wind-assisted In_2_S_3_ (P-In_2_S_3_) with a C–A interface is obtained. Compared to bare In_2_S_3_ nanosheets, there is a slight difference in the surface gloss of the P-In_2_S_3_ nanosheets.

**Figure 2:**
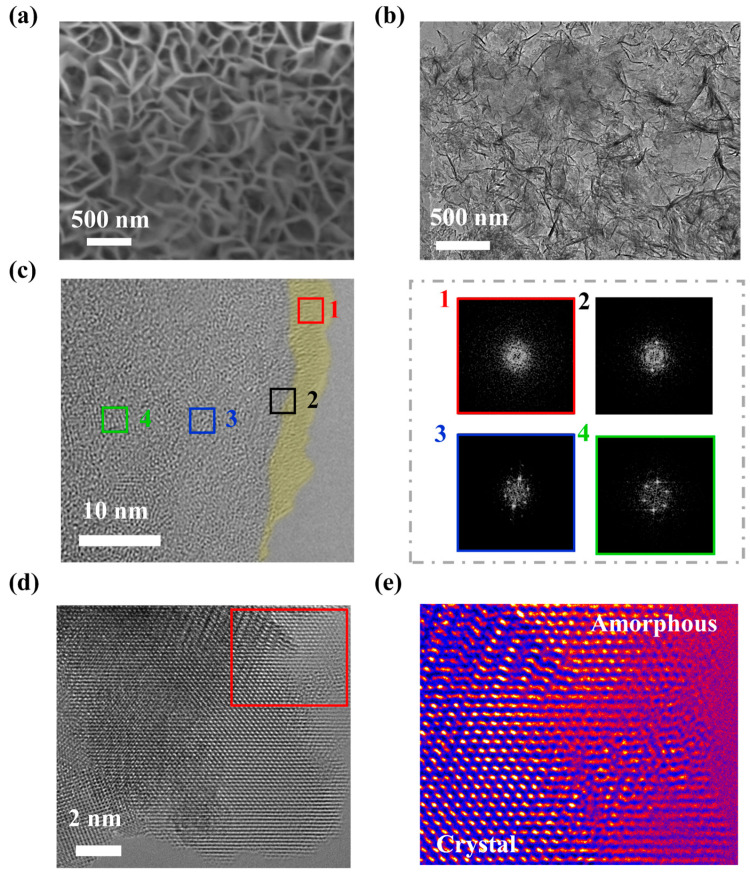
(**a**) The SEM image, (**b**) the TEM image, (**c**) the HRTEM image of the P-In_2_S_3_ sample. The Fourier transforms of the image involving the four box areas are displayed. (**d**) the HRTEM image of the P-In_2_S_3_ sample, (**e**) the filtered image of the red square area in (**d**).

Both samples are measured for further analyzing the surface structure. As shown in Figure S1, the morphology and the crystalline structure of the In_2_S_3_ sample are displayed, and the nanosheets grow vertically on the substrate. Figure 2 illustrates the morphology and structure of the P-In_2_S_3_ sample. Similarly, the SEM image in Figure 2a shows that the P-In_2_S_3_ sheets are vertically distributed on the substrate. As shown in Figure 2b, the nanosheets are tangled with the black lines on the nanosheet edges, most notably the gradually structural changing of the crystalline–amorphous (C–A) interface in Figure 2c. The four box areas in Figure 2c show Fourier transforms. Witness how the demonstrating microstructure changes from box 1 to box 4. Furthermore, the HRTEM and the related filtered images (Figure 2d,e) show that the lattice fringes are inconsecutive, and some of the lattice fringes are ambiguous, indicating the existence of an amorphous region.

**Figure 3:**
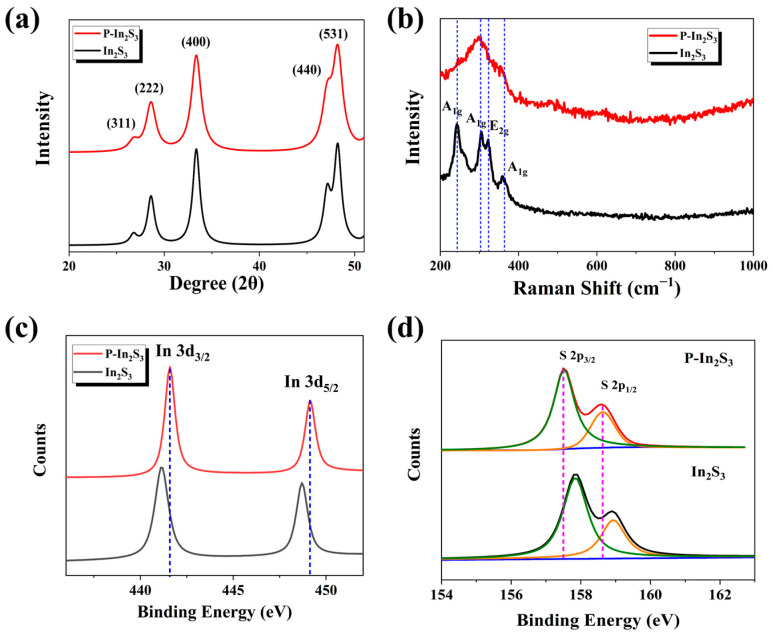
(**a**) The XRD patterns, (**b**) the Raman spectra, (**c**) the high-resolution XPS of element In, and (**d**) the high-resolution XPS of element S of the In_2_S_3_ and P-In_2_S_3_ samples.

To obtain the structural information, X-ray diffraction (XRD), Raman, and X-ray spectroscopy (XPS) are used to analyze the samples. In Figure 3a, the XRD patterns of both In_2_S_3_ and P-In_2_S_3_ nanosheets are indexable with the standard powder diffraction card #84-1385. The peaks of the bare In_2_S_3_ nanosheets located at 2θ degrees of 27.2°, 28.5°, 33.5°, 47.1°, and 48.2° are attributed to crystal faces (311), (222), (400), (440), and (531), respectively. After the plasma treatment, there is little change in the crystal face position but the weakened intensity of the crystal face (440), exhibiting robust long-range lattice arrangement and short-range structure conversion. As shown in Figure 3b, the different vibration modes, A_1g_ and E_2g_ of In_2_S_3,_ are d, with the related Raman peaks located at 244.23, 305.96, 320.69, and 360.40 cm^−1^, respectively [30,31]. After plasma treatment, the Raman vibration modes have been heavily altered. The Raman vibration modes are affected by local atomic arrangement, including factors such as stress, defects, and structural disorder [31], thus exhibiting the partial conversion of the atomic structure.

The XPS spectra further explore the conversion of crystal structure due to plasma treatment. Figure 3c shows high-resolution XPS spectra of elemental indium from samples. The In 3d_3/2_ and 3d_5/2_ peaks at 441.1 and 448.7 eV for the bare In_2_S_3_ nanosheets red-shift to 441.6 and 449.2 eV for the P-In_2_S_3_ nanosheets, respectively [32]. The red-shift of In binding energy shows that the electron cloud density of In atoms is reduced to indicate the coordination structure variation. Additionally, the sulfur’s 2p_3/2_ and 2p_1/2_ peak locations at 157.8 and 158.9 eV show a b (Figure 3d), indicating the electron cloud density around the S atom has increased [33,34], which would favor the hydrogen adsorption. All these measurements show that the atomic structure of In_2_S_3_ nanosheets can be altered through the plasma treatment, confirming the C–A interface present in the P-In_2_S_3_ nanosheets.

### 3.2. The Photocatalytic Hydrogen Production Performance

**Figure 4:**
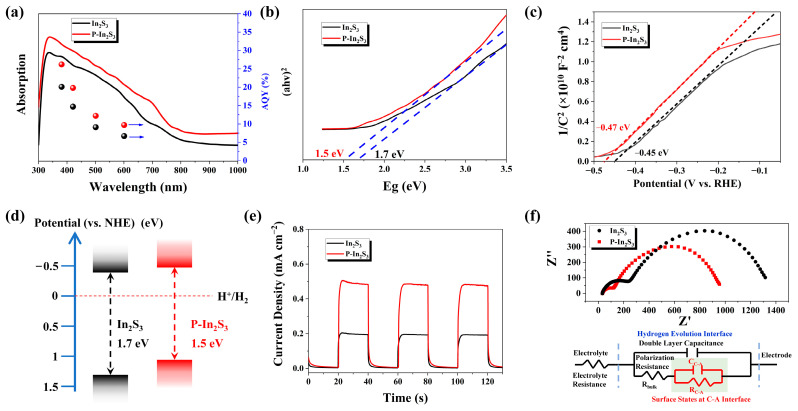
(**a**) The light absorption spectra and AQY, (**b**) the bandgap calculation based on the light absorption spectra, (**c**) the Mott–Schottky curves, (**d**) the schematic of energy band positions, (**e**) the current density versus time, and (**f**) the EIS curves of the In_2_S_3_ and P-In_2_S_3_ samples. The inset is the fitting resistance and capacitance diagram.

The photocatalytic reaction activity is mainly controlled by optical properties, electronic structure, and catalytic reactivity. First, as shown in Figure 4a, the light absorption spectra of the two samples have been measured, and the P-In_2_S_3_ sample has better light absorption compared with bare In_2_S_3_. This implies that plasma treatment is an effective way of improving photocatalyst optical absorption. The highest apparent quantum yield (AQY) of bare In_2_S_3_ is just 20.1% at 380 nm, whereas the AQY of P-In_2_S_3_ at 380 nm and 420 nm is 26.2% and 19.8%, respectively.

As for electronic properties, as shown in Figure 4b, the bandgap (E_g_) of these two materials can be calculated based on the UV-vis light absorption spectra. The bandgap of bare In_2_S_3_ is about 1.7 eV; it is about 1.5 eV for P-In_2_S_3_, which is beneficial to the use of visible light in the catalytic reaction. The Mott–Schottky (MS) curves of the samples are used to evaluate the carrier density (Figure 4c) preliminarily. The MS curve slope of P-In_2_S_3_ is larger than that of bare In_2_S_3_, indicating that more charge carriers exist in the P-In_2_S_3_ sample under illumination. As a result of the positive values of these MS slopes, the two samples behave as n-type semiconductors [35]. Considering that the conduction band is near the flat potential in an n-type semiconductor, the conduction bands of P-In_2_S_3_ and bare In_2_S_3_ are estimated to be −0.47 eV and −0.45 eV, respectively. Based on this analysis, the energy band structure can be summarized in Figure 4d, where the conduction band of P-In_2_S_3_ is more negative to the H^+^/H_2_ potential than that of In_2_S_3_, favoring the hydrogen production reaction in water molecules splitting. The changing trend of electron structure is also supported by the theoretical calculations (Figures S2 and S3).

The optical–electrical property is also mined through the photocurrent density, as shown in Figure 4e. The photocurrent density of P-In_2_S_3_ is about 0.48 mA cm^−2^, which is 2.4 times higher than that of In_2_S_3_ (0.21 mA cm^−2^). Such high photocurrent density results in a highly effective hydrogen production reaction. The charge separation efficiency (*η_sep_*) of the sample is calculated based on the equation

**Figure 5:**
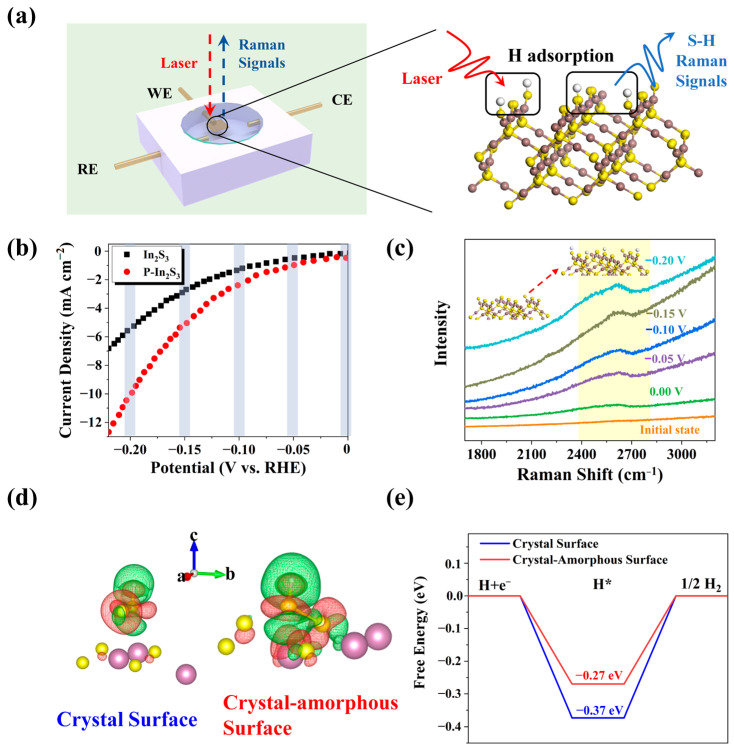
(**a**) The schematic of the operando Raman measurement of the samples. The RE, WE, and CE are the reference electrode, working electrode, and counter electrode, respectively. (**b**) LSV curves of the samples during the operando Raman measurement, (**c**) potential impact on the S–H bond Raman intensity during the operando Raman measurement of P-In_2_S_3_. The inset scheme is the hydrogen adsorption process of P-In_2_S_3_. (**d**) differential charge of hydrogen adsorption on the crystal surface and crystal–amorphous surface structures. The yellow spheres are S atoms, and the purple spheres are In atoms. The white sphere is the adsorption H atom. (**e**) Gibbs free energy of the hydrogen adsorption.

(1) ηsep=JsulfiteJabs

where *J_sulfite_* is the photocurrent density with the addition of Na_2_SO_3_ in the electrolyte solution, *J_abs_* is the photocurrent density when the adsorbed photons completely convert to currents. The calculated process is presented in supporting information [36,37]. The photocurrent densities *J_sulfite_* of the In_2_S_3_ and P-In_2_S_3_ samples are measured to be 0.21 and 0.48 mA cm^−2^, as shown in Figure 5e, and the *J_abs_* of the In_2_S_3_ and P-In_2_S_3_ samples are calculated to be 0.51 and 0.85 mA cm^−2^, respectively. Thus, the *η_sep_* values of the In_2_S_3_ and P-In_2_S_3_ are 41.3 % and 56.2 %, respectively, exhibiting that the plasma treatment can regulate the electronic structure to accelerate the separation of the charge carriers. In addition, the electrochemical impedance curves of the P-In_2_S_3_ and bare In_2_S_3_ samples are measured and shown in Figure 4f, where a smaller arc radius is observed for the P-In_2_S_3_ sample, implying faster interfacial electron transfer. The equivalent circuit of the impedance curve fitting of the resistance–capacitance scheme is displayed, and the addition of the C–A interface resulting from plasma treatment plays a crucial role in boosting electron transfer.

The active electrochemical area is a significant indication of the catalytic reaction property of the sample. As shown in Figure S4, the electrochemical surface area in bare In_2_S_3_ is about 3.62 mF cm^−2^, whereas, with P-In_2_S_3,_ the electrochemical surface area is 10.88 mF cm^−2^, which favors hydrogen adsorption in a catalytic reaction.

Moreover, the hydrogen adsorption process is further explored through the operando Raman measurement displayed in Figure 5a and Figure S5. Both bare In_2_S_3_ and P-In_2_S_3_ samples are deposited on the FTO as the working electrode. The counter and reference electrodes are platinum and silver/silver chloride (saturated potassium chloride electrolyte). After hydrogen adsorption to the active regions of the catalyst surface, intermediate bonds can form and be detected by Raman. At the same time, linear sweep voltammetry curves are measured to monitor the catalytic reaction stage, as shown in Figure 5b. As potential increases in value, the current density also increases in value. At the same potential, the current density of P-In_2_S_3_ is higher than that of bare In_2_S_3_, indicating more reactive electrons in the P-In_2_S_3_ sample. At different potentials, specifically −0.05, −0.10, −0.15, and −0.20 V, Raman spectra are collected to analyze hydrogen adsorption in the catalytic reaction (Figure 5c and Figure S6). The Raman peak at 2611 cm^−1^ is attributed to the S−H Raman vibration [38], which implies that the active sites are the sulfur atoms to adsorb the hydrogen atoms. Figure 5c shows that S−H Raman intensity rises with increased potential, suggesting that more hydrogen adsorption can be realized at a relatively high potential range. Importantly, it is noticed that the S−H Raman intensity of P-In_2_S_3_ is higher than that of bare In_2_S_3_ at any given potential, indicating that more hydrogen atoms are adsorbed onto the P-In_2_S_3_ surface during the catalytic reaction process compared with that of bare In_2_S_3_ (Figure S7).

The theoretical calculation is applied to analyze the catalytic performance of hydrogen adsorption (Figure 5d and Figure S8). About 0.373 (e) electron transfer on the amorphous surface, which is larger than that on the crystal surface (0.282 (e)). The Gibbs free energy of the hydrogen adsorption is calculated in Figure 5e, and a value closer to zero corresponds to better hydrogen adsorption performance [39]. On the crystal–amorphous surface, the free energy of the hydrogen adsorption is −0.27 eV, which is closer to zero than that on the crystal surface (−0.37 eV) in value (i.e., the absolute value of free energy of the hydrogen adsorption on the crystal–amorphous surface is smaller than that on the crystal surface), indicating that the crystal–amorphous surface favors the hydrogen adsorption.

**Figure 6:**
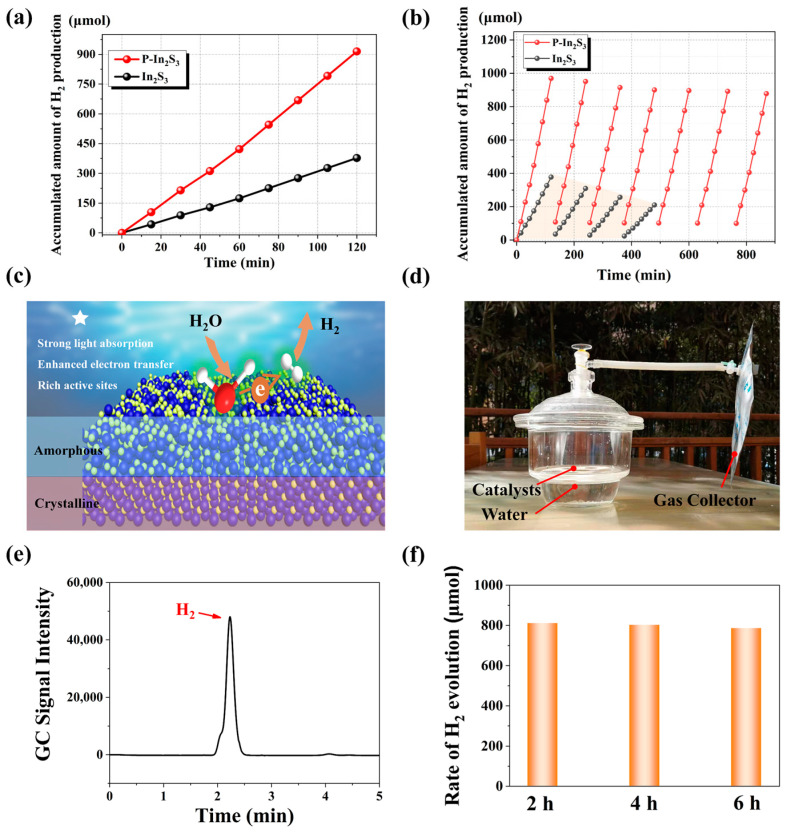
(**a**) accumulated amount of hydrogen production of different samples, (**b**) seven photocatalytic hydrogen production cycles of the P-In_2_S_3_ vs. four cycles of the bare In_2_S_3_, (**c**) the schematic of the photocatalytic hydrogen evolution reaction of the P-In_2_S_3_, (**d**) the photo of the P-In_2_S_3_ photocatalytic system, (**e**) the GC signal of the gas collector after 2 h collection, and (**f**) the rate of H_2_ evolution of the P-In_2_S_3_ photocatalytic system during the outdoor measurement.

Photocatalytic hydrogen production is measured with different samples, as shown in Figure 6a. The sacrificial agents of 0.3 M Na_2_S and 0.3 M Na_2_SO_3_ are used to capture photo-induced holes. At the end of a 120 min test, the amount of hydrogen production with bare In_2_S_3_ is about 337.5 μmol, and the average hydrogen production rate is calculated to be 168.60 μmol cm^−2^ h^−1^. In comparison, hydrogen production with P-In_2_S_3_ is about 914.7 μmol after 120 min, and the average hydrogen production rate is estimated to be 457.35 μmol cm^−2^ h^−1^ (the mass catalyst: 133 mg, converted rate 3438.72 μmol h^−1^ g^−1^). The comparison of the measurement data from this work with other references, including the hydrogen production rate, current density, and bandgap, has been made in Table S1 in the supporting information, exhibiting that the photocatalytic performance of P-In_2_S_3_ is dominant in these In_2_S_3_-based catalysts. These results have shown that plasma treatment can substantially enhance photocatalytic hydrogen production from water. It is easily observed from Figure 6b that the level of hydrogen production after the seventh test (about 840 min) is slightly smaller than that of the initial stage for P-In_2_S_3_, which is in sharp contrast to the catalytic performance of bare In_2_S_3,_ which shows drastic reduction of hydrogen production after only four test cycles, exhibiting high stability in the photocatalytic reaction of P-In_2_S_3_, also reflected by the morphology and structure of the samples (Figures S9 and S10).

A schematic of the photocatalytic hydrogen production reaction using P-In_2_S_3_ is shown in Figure 6c. After the plasma treatment of bare In_2_S_3_, the atomic arrangement of the nanosheets is altered to form an amorphous surface layer based on the bombardment from the plasma gas [40,41,42]. The amorphous region in the In_2_S_3_ nanosheets creates an unsaturated coordination environment to tune the electron structure. Therefore, P-In_2_S_3_ nanosheets with a C–A interface can improve light absorption and electron transfer. Moreover, the unsaturated coordination environment from the amorphous region endows the electrons gathering around S atoms, providing rich active sites for the catalytic reaction, thus leading to a highly efficient hydrogen production reaction.

A practical demo of the P-In_2_S_3_ photocatalytic system to satisfy real-world conditions is shown in Figure 6d. The P-In_2_S_3_ catalysts are laid on the surface of a perforated diaphragm, and the photocatalyst construction is immersed in water with the sacrificial agent. Owing to the transparency of the reactor, natural sunshine can illuminate the catalytic system. The reaction chamber outlet is connected to a gas collector, and the collected gas is detected through gas chromatography (GC) every two hours. A molecular hydrogen (H_2_) GC signal is detected, showing that the photocatalytic reaction works under natural outdoor conditions (Figure 6e). Three two-hour measurements are also shown in Figure 6f with a rate of H_2_ production of about 400.96 μmol cm^−2^ g^−1^ at the first 2 h, and the production rate of 400.50 μmol cm^−2^ g^−1^ after the 6 h test, indicating excellent stability.

## 4. Conclusions

We have designed and demonstrated a plasma-wind strategy for preparing In_2_S_3_ with an amorphous surface. We can achieve a photocatalytic water-splitting hydrogen production rate of 457.35 μmol cm^−2^ h^−1^ with high stability. Such impressive performance is due to the C–A interface, which improves light absorption and boosts charge carrier transfer to gather photo-induced electrons on the catalyst surface better. More importantly, the surface amorphous structure can cause an unsaturated coordination environment and gather electrons around the S atoms. In addition, the active reaction sites can be enriched as a result of the disordered atomic structure. This C–A interface can effectively balance catalytic activity and stability. Extending the P-In_2_S_3_ photocatalytic system under natural light conditions, a catalytic hydrogen production rate of 400.50 μmol cm^−2^ g^−1^ has been achieved, hopefully taking photocatalytic water splitting to a whole new level in practical energy applications.

## Data Availability

Data presented in this article is available on request from the corresponding author.

## Supplementary Materials

The following supporting information can be downloaded at: https://www.mdpi.com/article/10.3390/nano12101761/s1, Figure S1: (a) The SEM image, (b) the TEM image, and (c) the HRTEM image of the In_2_S_3_ sample. The inset is the Fourier transform of the black box area, and (d) the magnified HRTEM image of the In_2_S_3_ sample. The inset is the diffraction pattern of the sample; Figure S2: The material structures from the theoretical calculations. The yellow spheres are S atoms, and the purple spheres are In atoms; Figure S3: (a) The band structure of the crystal In_2_S_3_, (b) the band structure of the crystal–amorphous In_2_S_3_; Figure S4: (a) Electrochemical active area of the In_2_S_3_. The inset is the CVs of the In_2_S_3_ with different scan rates and (b) the Electrochemical active area of the P-In_2_S_3_. The inset is the CVs of the P-In_2_S_3_ with different scan rates; Figure S5: The photo of the operando Raman measurement; Figure S6: The potential impact on the operando Raman intensity of the bare In_2_S_3_ sample; Figure S7: The S–H bond Raman intensity after background subtraction versus the different potential during the operando Raman measurement; Figure S8: The hydrogen adsorption on the crystal surface and crystal–amorphous surface. The yellow spheres are S atoms, and the purple spheres are In atoms. The white sphere is the adsorption H atom; Figure S9: The morphology of the sample after the catalytic reaction. (a) bare In_2_S_3_, (b) P-In_2_S_3_. It is noticed that the surface of the bare In_2_S_3_ has been destroyed through the HRTEM imaging; comparatively, the structure of the P-In_2_S_3_ is well maintained; Figure S10: The Raman spectra of the P-In_2_S_3_ sample before and after catalytic reaction; Table S1: The comparison of hydrogen production rate, morphology, current density, and bandgap. References [24,25,43,44,45,46,47,48] are cited in the supplementary materials.
