# Supplementary material for: Plasma-Wind-Assisted In2S3 Preparation with an Amorphous Surface Structure for Enhanced Photocatalytic Hydrogen Production
Source: Nanomaterials (Basel). 2022 May 21;12(10):1761. doi: 10.3390/nano12101761 (PMC9143137; doi:10.3390/nano12101761)
Supplement: Supplementary file 1 [file nanomaterials-12-01761-s001.zip › nanomaterials-1723742-supplementary.pdf]

nanomaterials

MDPI

Supplementary Materials

Plasma-Wind-Assisted In_2S_3 Preparation with an Amorphous Surface Structure for Enhanced Photocatalytic Hydrogen Production

Shaohui Guo [1], Hui Luo [2], Xiaochuan Duan [1], Bingqing Wei [3,*] and Xianming Zhang [4,*]

[1] College of Chemistry, Taiyuan University of Technology, Taiyuan 030024, China; guoshaohui@tyut.edu.cn (S.G.); duanxiaochuan@tyut.edu.cn (X.D.)
[2] Department of Chemical Engineering, Imperial College London, South Kensington Campus, London SW7 2AZ, UK; hui.luo@imperial.ac.uk
[3] Department of Mechanical Engineering, University of Delaware, Newark, DE 19716, USA
[4] Key Laboratory of Interface Science and Engineering in Advanced Material, Ministry of Education, Taiyuan University of Technology, Taiyuan 030024, China
* Correspondence: weib@udel.edu (B.W.); zhangxianming@tyut.edu.cn (X.Z.)

S1. Calculation of the Theoretical Photocurrents J_{abs} of the Samples

The single photo energy is calculated from the following Equation:

$$E(\lambda) = h * \frac{C}{\lambda} \tag{S1}$$

where $E(\lambda)$ is the photo energy (J), h is the Planck's constant (6.626 × 10^{-34} J s^{-1}), C is the speed of light (3 × 10^8 m s^{-1}), and λ is the photon wavelength (nm).

The solar photon flux is calculated from the following Equation:

$$Flux(\lambda) = \frac{P(\lambda)}{E(\lambda)} \tag{S2}$$

where $Flux(\lambda)$ is the solar photon flux, and $P(\lambda)$ is the solar power flux (Reference Solar Spectral Irradiance: Air Mass 1.5. NREL. ASTM G-173 package. http://rredc.nrel.gov/solar/spectra/am1.5/ (accessed on 30 November 2021)).

The theoretical maximum photocurrent density under solar illumination, J_{abs} is then calculated by integrating the solar photon flux as shown in the following Equation:

$$J_{abs} = e \times \int_{\lambda 2}^{\lambda 1} Flux(\lambda) d\lambda \tag{S3}$$

where $\lambda 1$ is the absorption edge of the sample according to Figure 4a, $\lambda 2$ is the lower limit of the measured solar spectrum, and e is the elementary charge (1.602×10^{-19} C). The theoretical photocurrents of the In_2S_3 and P-In_2S_3 are accordingly calculated to be 0.51 and 0.85 mA cm^{-2}, respectively.

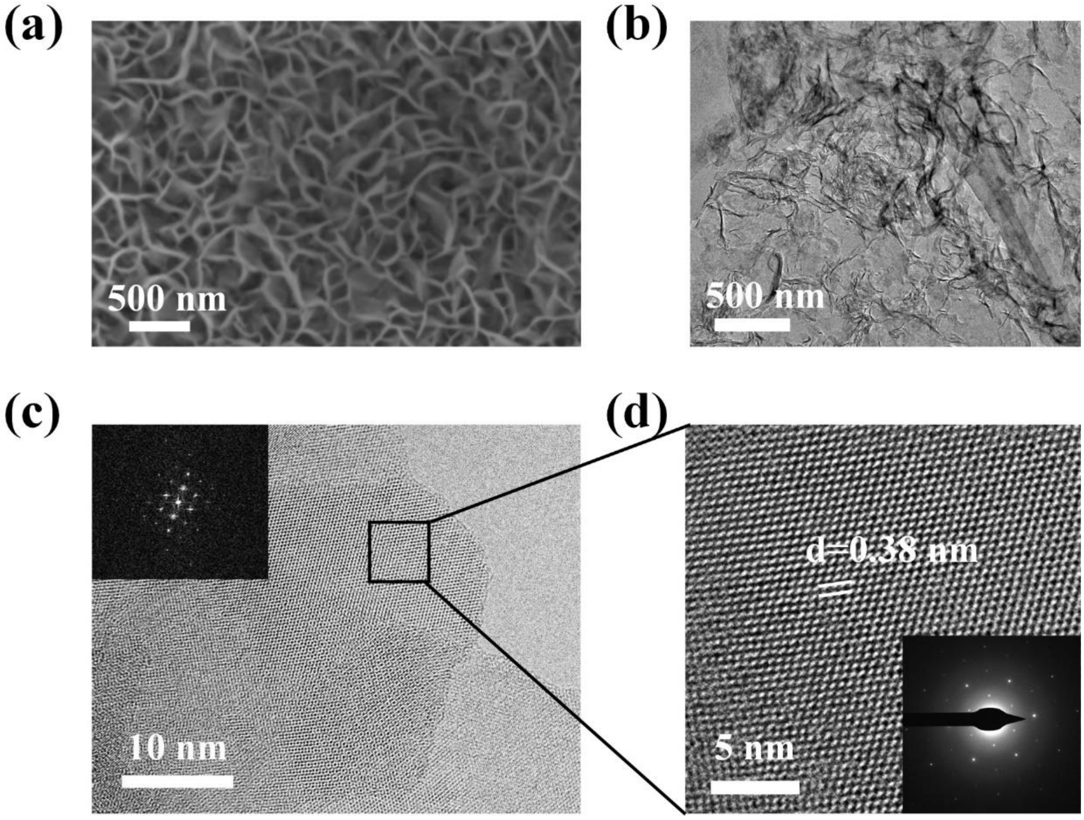

Figure S1. (**a**) The SEM image, (**b**) the TEM image, and (**c**) the HRTEM image of the In_2S_3 sample. The inset is the Fourier transform of the black box area, and (**d**) the magnified HRTEM image of the In_2S_3 sample. The inset is the diffraction pattern of the sample.

The morphology of a bare In_2S_3 nanosheet is shown in Figure S1. Figure S1a shows a scanning electron micrograph (SEM) image of the sample, showing In_2S_3 nanosheets grown vertically on an FTO substrate, and the clear lines are the nanosheet edges. In Figure S1b, it is easily seen that the nanosheets are twisted like silk threads. In the high-resolution transmission electron microscopy image (Figure S1c and S1d), the crystal lattices of the sample can be seen, and their clean edges are focused on illustrating that a good crystal structure can be obtained. This is confirmed by the diffraction pattern of the sample In_2S_3 shown in Figure S1d.

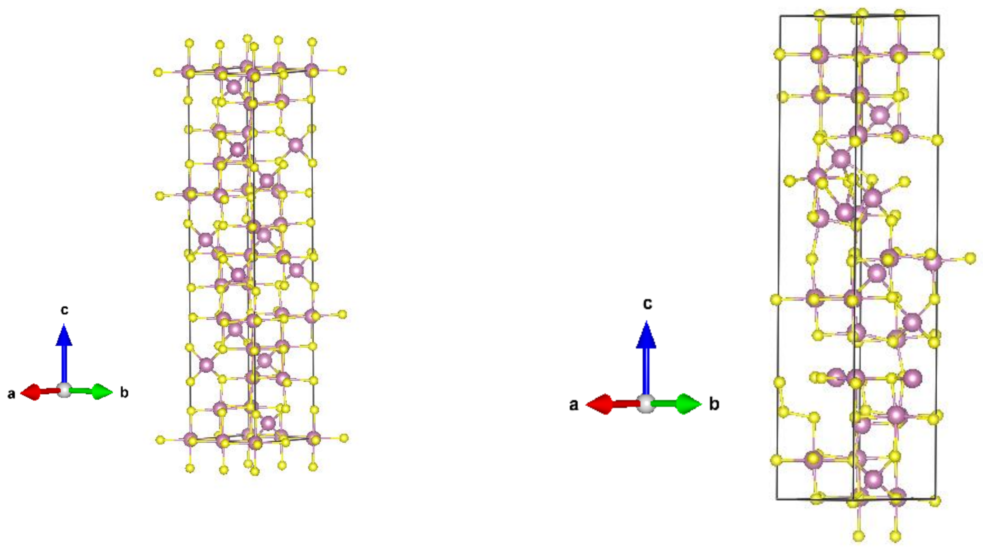

Crystal structure **Crystal-amorphous structure**

Figure S2. The material structures from the theoretical calculations. The yellow spheres are S atoms, and the purple spheres are In atoms.

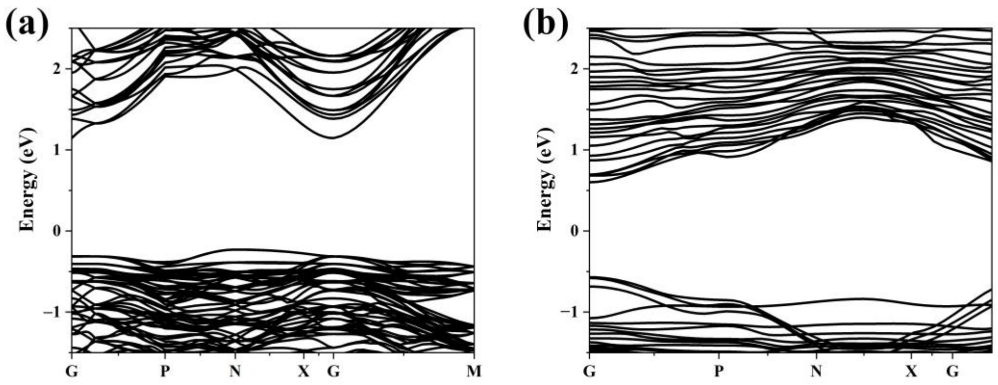

Figure S3. (**a**) The band structure of the crystal In_2S_3, (**b**) the band structure of the crystal-amorphous In_2S_3.

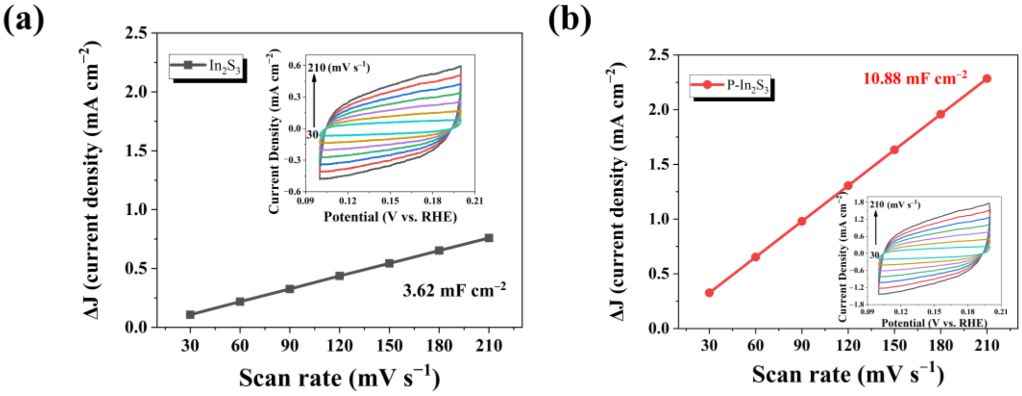

Figure S4. (**a**) Electrochemical active area of the In_2S_3. The inset is the CVs of the In_2S_3 with different scan rates and (**b**) the Electrochemical active area of the P-In_2S_3. The inset is the CVs of the P-In_2S_3 with varying scan rates.

In Figures S4a and S4b, the active electrochemical areas of the samples have been measured via the method of capacitive currents and different scan rates, where the scan rate varies from 30 mV s^{-1} to 210 mV s^{-1}. The electrochemical surface area in bare In_2S_3 is about 3.62 mF cm^{-2}, whereas, with P-In_2S_3, the electrochemical surface area is 10.88 mF cm^{-2}, which favors hydrogen adsorption in a catalytic reaction.

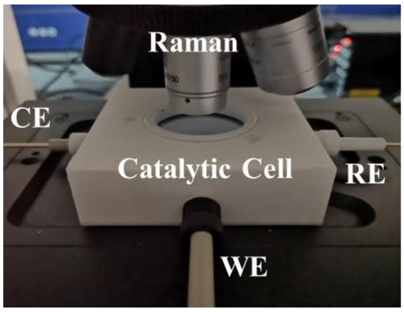

Figure S5. The photo of the operando Raman measurement.

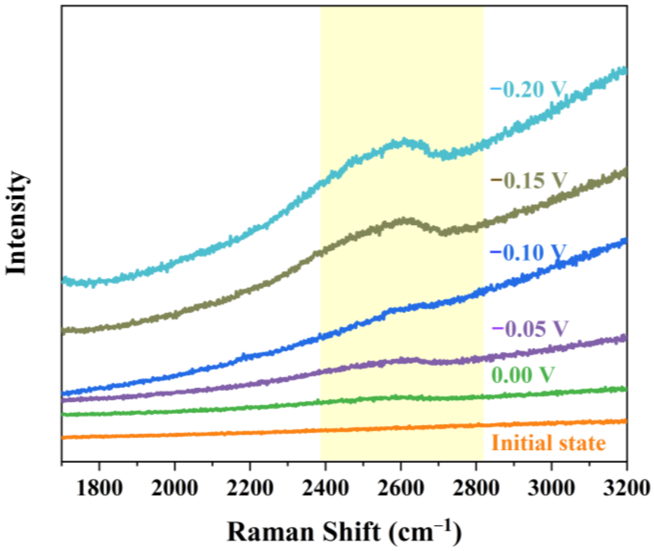

Figure S6. The potential impact on the operando Raman intensity of the bare In_2S_3 sample.

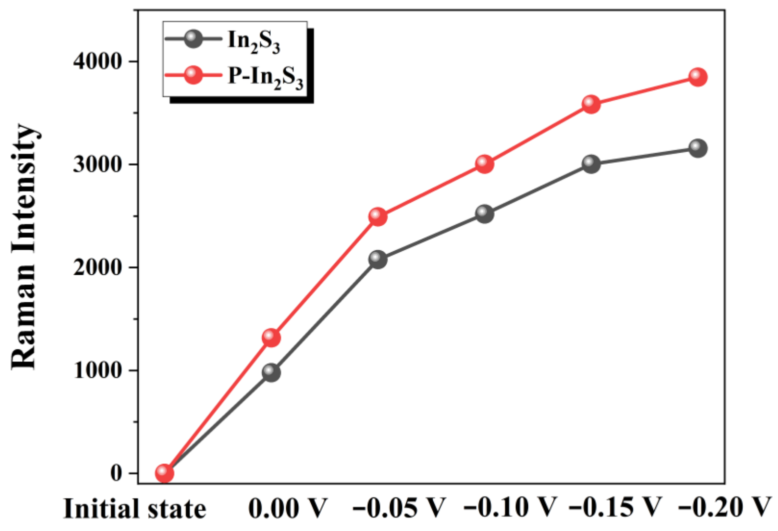

Figure S7. The S–H bond Raman intensity after background subtraction versus the different potential during the operando Raman measurement.

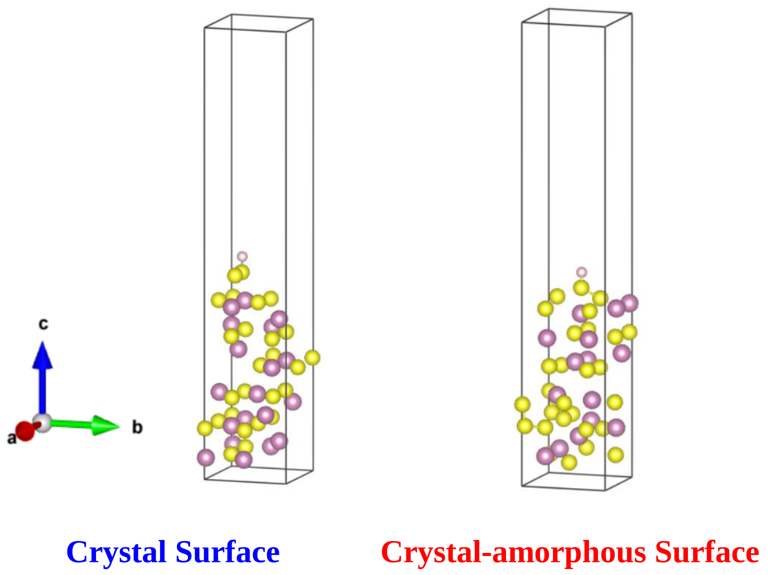

Figure S8. The hydrogen adsorption on the crystal surface and crystal-amorphous surface. The yellow spheres are S atoms, and the purple spheres are In atoms. The white sphere is the adsorption H atom.

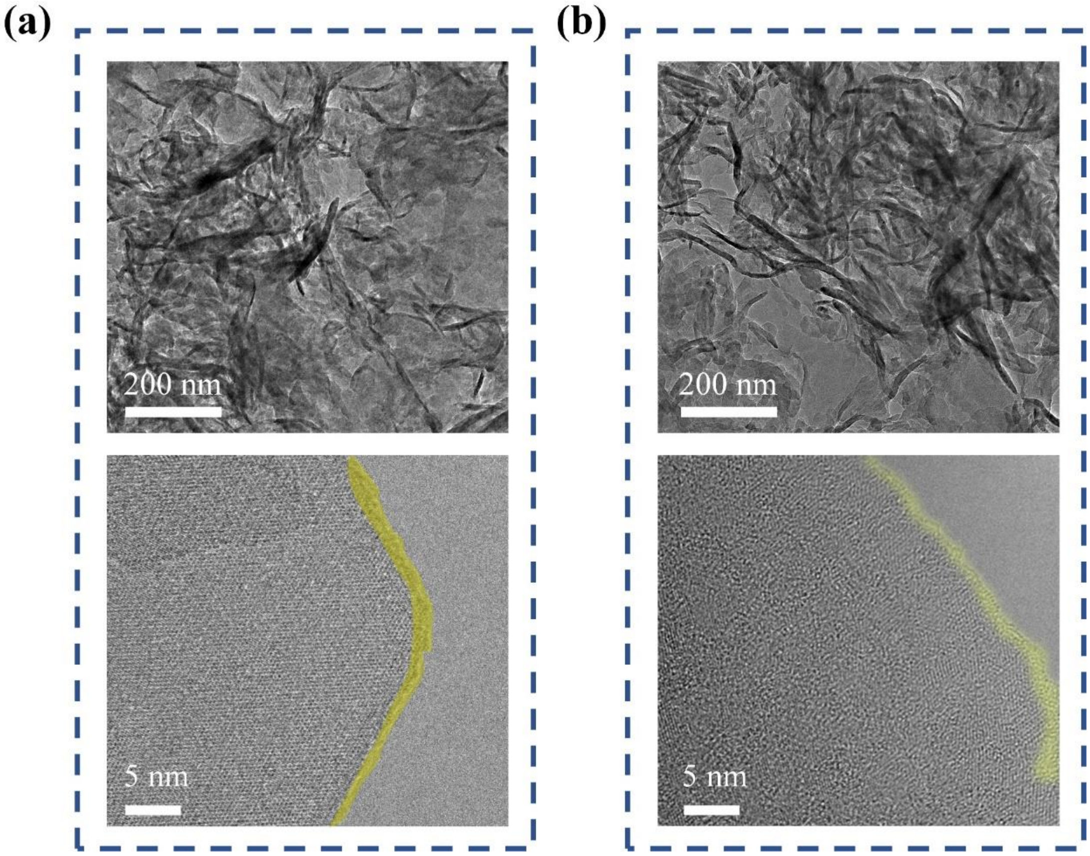

Figure S9. The morphology of the sample after the catalytic reaction. (**a**) bare In_2S_3, (**b**) P-In_2S_3. It is noticed that the surface of the bare In_2S_3 has been destroyed through the HRTEM imaging; comparatively, the structure of the P-In_2S_3 is well maintained.

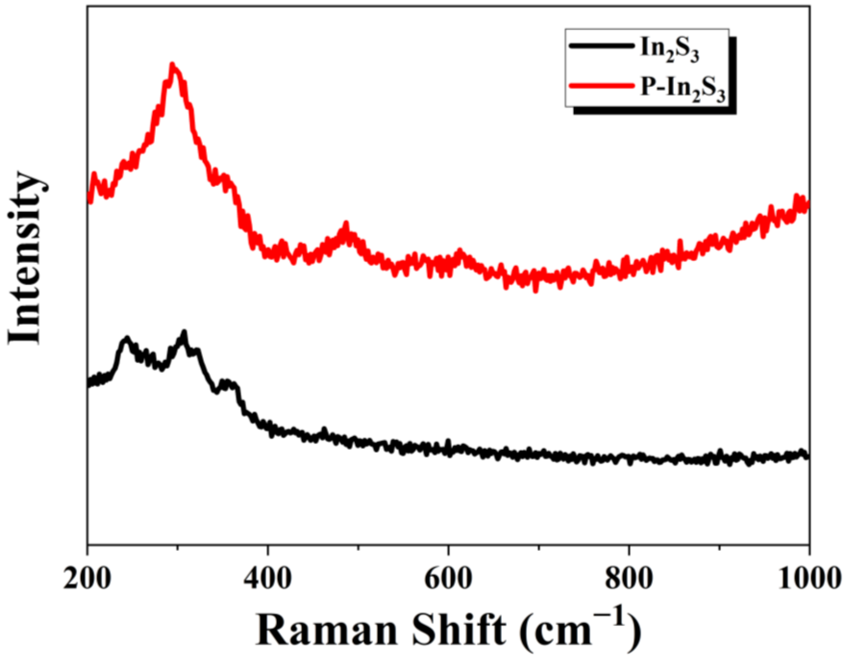

Figure S10. The Raman spectra of the P-In_2S_3 sample before and after the catalytic reaction.

Table S1. The comparison of hydrogen production rate, morphology, current density, and bandgap.

Sample	Rate of H_2 Production	Morphology	Current Density	Bandgap	References
P-In_2S_3	457.35 μmol cm^{-2} h^{-1} (3423.14 μmol h^{-1} g^{-1})	surface amorphous nanosheets	0.48 mA cm^{-2}	1.5 eV	this work
MoS_2@In_2S_3/Bi_2S_3	973.42 μmol h^{-1} g^{-1}	core-shell heterojunction	9 μA cm^{-2}	-	[1]
$ZnIn_2S_4$-MIL-68/In_2S_3	306 μmol h^{-1} g^{-1}	nanoparticles on microtubes	0.64 μA cm^{-2}	2.38 eV	[2]
MoP/In_2S_3	481.73 μmol h^{-1} g^{-1}	heterojunction	1.7 μA cm^{-2}	2.01 eV	[3]
Mo_2C-In_2S_3	535.58 μmol h^{-1} g^{-1}	heterojunction	1.5 μA cm^{-2}	-	[4]
In_2S_3-WC	390.52 μmol h^{-1} g^{-1}	flowers heterojunction	1.8 μA cm^{-2}	1.88 eV	[5]
GO/Fe2P/In_2S_3	483.35 μmol h^{-1} g^{-1}	flowers heterojunction	-	-	[6]
In_2S_3/$ZnIn_2S_4$	12.1 μmol cm^{-2} h^{-1}	nanosheets heterojunction	1.06 mA cm^{-2}	2.0 eV	[7]
$Zn_mIn_2S_{m+3}$@In_2S_3	3300 μmol h^{-1} g^{-1}	flowers heterojunction	90 μA cm^{-2}	-	[8]

References

1. Wang, Y.; Xing, Z.; Zhao, H.; Song, S.; Liu, M.; Li, Z.; Zhou, W. MoS_2@In_2S_3/Bi_2S_3 Core-shell dual Z-scheme tandem heterojunctions with Broad-spectrum response and enhanced Photothermal-photocatalytic performance. *Chem. Eng. J.* **2022**, *431*, 133355, https://doi.org/10.1016/j.cej.2021.133355.
2. Song, H.; Zhang, Q.; Hu, D.; Sun, Z.; Han, Y.; Meng, H.; Sun, T.; Zhang, X. In-situ partial cation exchange-derived $ZnIn_2S_4$ nanoparticles hybridized 1D MIL-68/In_2S_3 microtubes for highly efficient visible-light induced photocatalytic H_2 production. *Sep. Purif. Technol.* **2022**, *287*, 120585, https://doi.org/10.1016/j.seppur.2022.120585.
3. Ma, X.; Li, W.; Li, H.; Dong, M.; Li, X.; Geng, L.; Fan, H.; Li, Y.; Qiu, H.; Wang, T. Fabrication of novel and noble-metal-free MoP/In_2S_3 Schottky heterojunction photocatalyst with efficient charge separation for enhanced photocatalytic H_2 evolution under visible light. *J. Colloid Interface Sci.* **2022**, *617*, 284–292, https://doi.org/10.1016/j.jcis.2022.03.021.
4. Ma, X.; Ren, C.; Li, H.; Liu, X.; Li, X.; Han, K.; Li, W.; Zhan, Y.; Khan, A.; Chang, Z.; et al. A novel noble-metal-free Mo_2C-In_2S_3 heterojunction photocatalyst with efficient charge separation for enhanced photocatalytic H_2 evolution under visible light. *J. Colloid Interface Sci.* **2020**, *582*, 488–495, https://doi.org/10.1016/j.jcis.2020.08.083.
5. Ma, X.; Li, W.; Ren, C.; Li, H.; Liu, X.; Li, X.; Wang, T.; Dong, M.; Liu, S.; Chen, S. A novel noble-metal-free binary and ternary In2S3 photocatalyst with WC and "W-Mo auxiliary pairs" for highly-efficient visible-light hydrogen evolution. *J. Alloy. Compd.* **2021**, *875*, 160058, https://doi.org/10.1016/j.jallcom.2021.160058.
6. Li, X.; Lyu, X.; Zhao, X.; Zhang, Y.; Akanyange, S.N.; Crittenden, J.C.; Zhao, H.; Jiang, T. Enhanced photocatalytic H_2 evolution over In_2S_3 via decoration with GO and Fe_2P co-catalysts. *Int. J. Hydrogen Energy* **2021**, *46*, 18376–18390, https://doi.org/10.1016/j.ijhydene.2021.03.017.
7. Geng, H.; Ying, P.; Li, K.; Zhao, Y.; Gu, X. Epitaxial In_2S_3/$ZnIn_2S_4$ heterojunction nanosheet arrays on FTO substrates for photoelectrochemical water splitting. *Appl. Surf. Sci.* **2021**, *563*, 150289, https://doi.org/10.1016/j.apsusc.2021.150289.
8. Li, Y.; Han, P.; Hou, Y.; Peng, S.; Kuang, X. Oriented ZnmIn_2S_{m+3}@In_2S_3 heterojunction with hierarchical structure for efficient photocatalytic hydrogen evolution. *Appl. Catal. B: Environ.* **2018**, *244*, 604–611, https://doi.org/10.1016/j.apcatb.2018.11.088.
